# Double-Network Polysaccharide-Collagen Hybrid Bioink

**DOI:** 10.1021/acsami.5c20304

**Published:** 2025-12-07

**Authors:** Fabian Tribukait-Riemenschneider, V. Prasad Shastri

**Affiliations:** 1 Institute for Macromolecular Chemistry, 9174 University of Freiburg,Freiburg 79104, Germany; 2 Institute for Macromolecular Chemistry and BIOSS - Centre for Biological Signalling Studies, 9174 University of Freiburg, Freiburg 79104, Germany

**Keywords:** hybrid bioink, carboxylated agarose, collagen, 3D bioprinting, interpenetrating network

## Abstract

Over the past decade, three-dimensional bioprinting (3DBP) has evolved into a versatile processing tool for engineering tissues. The key component, the bioink, can be composed of many different hydrogel-forming polymers, which are mostly performant in either mechanical or biological properties but seldom both. Carboxylated agarose (CA) was combined here with collagen type 1 to simultaneously satisfy both properties and combine their attributes, without compromising on either; the result is an innovative, hybrid bioink. It can be printed with high accuracy, good layer-to-layer adhesion, and rapid gelation, enabling overhang printing. Scanning electron microscopy (SEM) and fluorescently labeled collagen demonstrated that the two components mixed well, resulting in uniformly distributed collagen fibrils and a double network. From the biological perspective, a bioink must exhibit cell-adhesion moieties to maintain proliferation and metabolism, which we could ensure through the collagen component. Here, we present an example strategy for combining an inert polysaccharide with bioactive collagen, two polymers with opposing gelation conditions, which yields a bioink that possesses beneficial properties from both without compromising the features of either. The interpenetrating structure of both molecules synergistically balances the mechanical strength of CA and the biological functionality of collagen.

## Graphical abstract

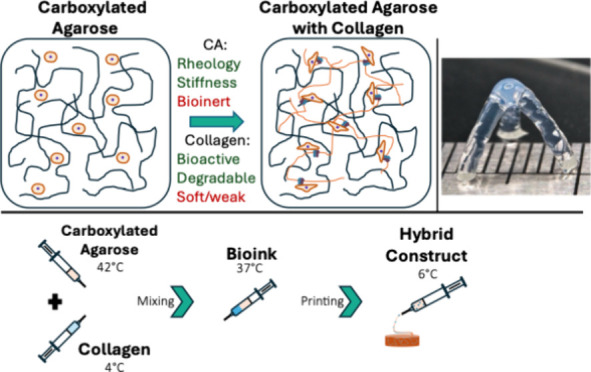

## 1 Introduction

Regenerative medicine and especially tissue engineering are constantly evolving fields aiming to create artificial tissues and structures that can replace or replicate healthy ones. A key task in this effort is the development of biomaterials that support and facilitate interactions between living cells and the construct.^1^ These materials are needed either as a matrix for cell seeding in engineered tissues or as nonliving implants intended for long-term integration within the patient’s body. In both scenarios, the nature of the interaction between cells and the material is of fundamental importance. One field, in particular, revolves around the design of novel materials and the application thereof in a biological context: 3D Bioprinting.^2–4^ 3D-bioprinting relies on “bioinks”, materials used to deliver biological information (cells, growth factors) in a spatially defined manner. Bioinks must possess adequate physical and biological properties, as they need to support the formation of stable 3D structures, withstand handling, and survive implantation, while also providing instructive cues to cells to coax their development into functional tissue.^5^ For clinical translation, bioinks should additionally possess an exceptional safety profile and meet regulatory requirements.

The physical requirements encompass the rheological behavior of the ink, which must support extrusion at acceptable pressures and yield a stable filament, and subsequently structures with defined and reproducible mechanical properties, since cells are strongly influenced by the mechanical characteristics of their microenvironment.^6–8^ An ideal bioink should exhibit shear-thinning, hold its own weight, preserve its printed form, and match the stiffness needed for the application.^9^ The biological requirements include the presence of cell-adhesion motifs, cytocompatibility, the ability to incorporate bioactive molecules, and a predictable biological response. Only materials that can meet several of these stringent demands qualify as bioinks.^10^ Achieving both the biological and physical requirements concurrently, i.e., in the same system, remains a significant challenge.

Most commonly used bioinks excel in either mechanical or biological functionality but show limited performance in the other. To address this trade-off, researchers tried to supplement bioinks with additives, modify polymers, or blend polymers of different classes. An established example of such an additive is laponite, a synthetic nanosilicate which has been shown to improve the rheological properties of collagen-based hydrogels, while simultaneously inducing osteogenic differentiation in mesenchymal stem cells (MSC).^11^ The underlying base hydrogel, gelatin methacrylate, illustrates polymer modification. The methacrylation creates cross-linking capabilities and thus mechanical stability to the normally brittle gelatin hydrogel that already supports cellular adhesion and cytocompatibility.^12^ However, the requirement of UV light for cross-linking negatively impacts cytocompatibility. Alternatively, blending two polymers that complement each other’s properties can be an option. For example, Gu et al. formulated a bioink by combining carboxylated agarose (CA), a material with strong mechanical properties, with gelatin. This approach successfully enhanced the biological performance of CA but was limited by the diffusion of gelatin over extended culture periods.^13^ Overall, these examples show the recurring problem: Most polymers provide either strong physical performance or strong biological performance, but seldom both. Addressing this limitation requires the combination of complementary materials to form hybrid hydrogels.

In this work, we explore such an approach by combining a CA-based bioink with a type 1 collagen solution. Polysaccharides such as CA are frequently considered promising for their rheological properties and mechanical stability, making them suitable for precise 3D printing.^14,15^ The CA system in particular has been shown to exhibit favorable rheological properties and rapid gelation, supporting accurate and straightforward printing, while its tunable nature allows adaptation to application-specific stiffness.^16–19^ However, from a biological perspective, carboxylated agarose is inert; while this results in low cytotoxicity, it also prevents cell-matrix interactions. Cells encapsulated in (carboxylated) agarose bioinks typically remain suspended, adopt a spherical morphology, and show limited proliferative activity.^13,18,20^ Collagen (type 1), by contrast, is expected to improve biological functionality, particularly cell adhesion and proliferation, without significantly compromising the mechanical and rheological benefits of CA (Figure 1A). Furthermore, the straightforward blending approach described here avoids the need for chemical modification or processing steps. Potential challenges were identified in the poor solubility of collagen type 1, which limits the maximum amount in a potential bioink composite. Moreover, CA and collagen present contrasting gelation mechanisms. CA undergoes physical gelation upon cooling below its gelation temperature, whereas collagen type 1 undergoes fibrillogenesis when warmed to physiological temperatures at neutral pH.^21^ By carefully controlling the mixing conditions, in this study, we report a homogeneous composite bioink that integrates the rheological advantages of CA with the biological benefits of type 1 collagen, i.e., cellular attachment (Figure 1B).

**Figure 1:**
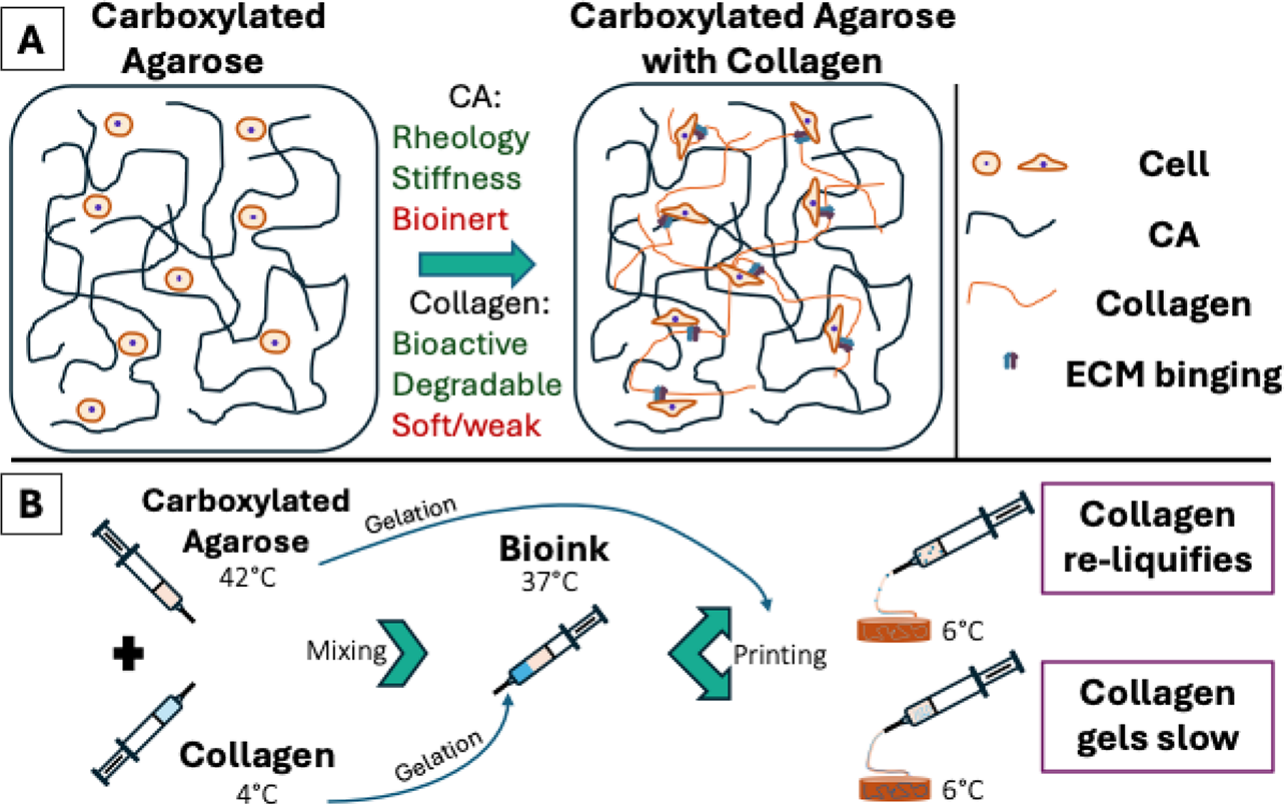
Schematic representation of the hybrid bioink composed of carboxylated agarose and collagen type 1 and its mixing strategy. (A) The bioink integrates the rheological and mechanical properties of CA with the biological functionality of collagen and thereby enables cellular attachment in the bioinert CA system. (B) The mixing strategy combines warm CA and cold collagen under controlled conditions, yielding a homogeneous bioink via one of two hypothesized mechanisms: Partial reliquification of preassembled collagen fibrils upon being cooled after extrusion, or slow fibrillogenesis of collagen within the CA network throughout the entire printing process.

## 2 Materials and Methods

### 2.1 Synthesis of Carboxylated Agarose

Native agarose was carboxylated as previously described.^19^ Briefly, 10 g of native agarose (NA) type 1 (GeneOn, Germany) and 100 mL of H_2_O were loaded into a three-necked round-bottom flask, with a stirrer and a pH meter. The mixture was brought to 90 °C to dissolve the agarose and then cooled down to 0 °C while stirring vigorously to prevent gelation. Afterward, 300 mg TEMPO (Abcr, Germany), 1.5 g NaBr, and 3.75 mL NaOCl (15%v/v) were added. The pH of the solution was monitored and kept at 10.8 throughout the entire reaction, and the degree of carboxylation was controlled by the addition of predetermined volumes of NaOH solution (0.5 M). To finalize the modification, 1.5 g NaBH_4_ was added, the pH was brought to 8, and the mixture was stirred for 1 h. The product was then precipitated by the addition of 150 g NaCl and 2 L of ethanol, after which the collected solid was vacuum filtered and extracted with ethanol. After extensive dialysis against water, the obtained carboxylated agarose was freeze-dried and stored in the fridge until use.

### 2.2 Rheological Characterization of Bioinks

All the rheological tests were performed using a Kinexus-PRO+ rotary rheometer with a stainless-steel geometry featuring a 20 mm diameter and a 1° cone. All samples were allowed to equilibrate to the testing temperature for at least 1 h before the measurements. All measurements were conducted in triplicate and averaged within OriginPro 2023. The temperature sweep was performed from 60 to 4 °C, and *G*′ and *G*″ were recorded.

### 2.3 Scanning Electron Microscopy and Critical Point Drying

Hydrogels for scanning electron microscopy (SEM) were printed and processed with a critical point drying (CPD) method to preserve their microscale structure as per a published protocol.^22^ Briefly, right after printing, the hydrogels were dehydrated with increasing concentrations of ethanol for 1 h each (50–100%). The dehydrated hydrogels were then transferred into the critical point drying (CPD) machine and immersed in biological-grade, 200-proof ethanol. The device was programmed to run slowly for 5 h and 30 min. Afterward, the gels were immediately mounted with double-sided carbon tape and sputter-coated with gold. For internal structures, they were immersed in liquid nitrogen for 20 s, carefully broken with tweezers, and then mounted and sputtered. The SEM imaging was conducted using a Quanta 250 FEG electron microscope (FEI, USA) with an acceleration voltage of 10 kV.

### 2.4 Computer-Aided Design and 3D Printing

All 3D models were designed in Autodesk Inventor Professional (2020–2024), exported as an .stl file, and then sliced in Heartware (Cellink). Gcode files were additionally edited manually to enhance printing behavior and ensure flawless printing. 3D printing of hydrogels was performed using a modified Cellink Inkredible+ in a sterile environment (laminar flow hood). The custom modifications include an aluminum nozzle heater and an aluminum print bed with integrated water cooling.

### 2.5 Carboxylated Agarose and Collagen Blend Bioink Preparation

For the printing of CA and CANA, the hydrogels were liquified in a water bath at 94 °C, then brought to 42 °C in a temperature-controlled brass cylinder for 10 min and loaded into a preheated metal syringe at 37 °C. After 10 min, the printing was started on the printbed, which was cooled to 6 °C. If cells were printed, the bioink was loaded with a highly concentrated cell suspension just before transferring the bioink into the metal syringe. If a collagen (type 1) solution was added to the ink, it was always added right before adding the cells to the 42 °C ink. In both cases, the bioink was briefly vortexed.

The pneumatic printing pressure was adjusted individually before each print to ensure a consistent and steady flow. Throughout the printing procedure, the pressure was further adjusted if needed to maintain a consistent and steady flow.

Printing workflow:

$$\mathrm{CA}\left(95^{{}^{\circ}}C\right)\to \mathrm{CA}\left(45^{{}^{\circ}}C\right)\overset{\mathrm{collagen}\left(4^{{}^{\circ}}C\right)}{\to }\mathrm{vortex}\overset{\mathrm{cells}\left(37^{{}^{\circ}}C\right)}{\to }\mathrm{vortex}\to \mathrm{finalink}\left(37^{{}^{\circ}}C\right)\overset{\mathrm{printing}}{\to }\mathrm{construct}\left(4^{{}^{\circ}}C\right)$$

For fluorescent microscopy of the hybrid hydrogel, FITC-conjugated collagen type 1 solution (Abcam, Germany) was mixed with CA using the same workflow. Afterward, the mixture was either dropped on a cooled microscopy slide and immediately coverslipped or cast into small cylinders (2.5 mm × 2.5 mm). The cylinders were embedded in OCT overnight and frozen. OCT-embedded cylinders were cut into 10 μm-thick sections using a cryotome (Zeiss Hyrax C20).

### 2.6 Cell Culture

Cells were thawed in a 37 °C water bath and immediately transferred into a culture vessel containing prewarmed media as soon as no more than a small ice crystal remained. Media was changed 2–3 times a week for all cells. All cells were cultured at 37 °C under 5% CO_2_ in their respective media. For passaging, cells at 70–80% confluency were washed with Dulbecco’s PBS (DPBS, Gibco, Germany) and trypsinized (0.05% trypsin/0.02% EDTA) for 5 min or until most of the cells detached. Cells were tested negative for mycoplasma, and HepG2 cells were genotyped before purchase.

HepG2 cells were procured from the BIOSS Toolbox (Signal Haus, University of Freiburg) and cultured in high glucose DMEM (4.5% glucose, Glutamax, and Sodium pyruvate) (Gibco, Germany) supplemented with 10% v/v fetal bovine serum (FBS) (Gibco, Germany). Human nasal chondrocytes (hNC) were isolated from the nasal cartilage of healthy donors. The tissue samples were obtained during orthopedic procedures under the general informed consent of the University Hospital Basel and in accordance with the local ethical committee’s regulations (University Hospital Basel, ref Number 78/07). hNC were expanded with chondrocyte expansion medium containing 87% DMEM with high glucose 4.5%, Glutamax and Sodium pyruvate (Gibco, Germany), 10% FBS (Gibco, Germany), 1% penicillin/streptomycin (PAN-Biotech, Germany), 1% 1 M HEPES (PAN-Biotech, Germany), 1% MEM-NEAA (100×, PAN-Biotech, Germany), and 1 ng/mL TGF-β1 (R&D systems), 5 ng/mL FGF-2 (R&D systems).

### 2.7 Metabolic Assays

#### 2.7.1 Blood Urea Nitrogen (BUN)

The BUN assay was performed following the kit manufacturer’s (Invitrogen) instructions. Briefly, media samples (without phenol red) were collected after the indicated time point and frozen immediately until measured. For 24 h before media collection, the cells were incubated with 6 mM NH_4_, added to the regular culture media. For the measurement, samples were mixed with the color reagents, incubated for 30 min, and then the absorbance was read at 450 nm in a Biotek Synergy-HT plate reader (Biotek Instruments, USA). The concentration was thereafter calculated using a standard curve with a known urea concentration.

#### 2.7.2 GAG and DNA Quantification

For the glycosaminoglycan (GAG)/DNA assay, printed constructs were lyophilized at the indicated time point, and their dry weight was measured. Each sample was digested in 500 μL 125 μg/mL papain cocktail solution (Sigma P3125, Germany) for 16 h at 60 °C. After that, the samples were centrifuged at 10,000 rpm for 10 min, and the supernatant was used for GAG and DNA analysis. The amount of GAG was quantified using dimethylmethylene blue (DMMB). Briefly, 100 μL of supernatant was transferred into 1 mL of DMMB solution and placed on a shaker for 30 min at room temperature. Subsequently, samples were centrifuged at 12,000 rpm for 10 min to sediment the GAG as a purple pellet. The supernatant was removed, and 700 μL of decomplexion solution was added to each pellet, and the samples were incubated at 60 °C for 20 min. The reacted mixture was then pipetted into a 96-well plate (200 μL/well), and the absorbance was measured at 656 nm using the plate reader. The GAG amount was calculated using a standard curve with a known GAG concentration. For DNA Quantification, 5 μL of the digested supernatant was diluted with 95 μL of 1× TE buffer. 1× TE buffer was prepared by dilution of 20× TE buffer in RNA/DNA-free water (Promega, USA). Then, 100 μL of the diluted sample solution was added to 100 μL Pico Green working solution (working concentration 5 μL/mL Pico Green (200×, Molecular Probes, USA)) in 1× TE buffer. Plates were incubated in the dark for 5 min, and fluorescence was measured using the plate reader with an excitation wavelength of 485 nm and an emission wavelength of 528 nm. The DNA concentration was calculated using a standard curve with a known DNA concentration.

### 2.8 Histology

Printed constructs were fixed, washed with DPBS, embedded in OCT, and then frozen. OCT-embedded blocks were cut into 7 μm-thick sections using a cryotome. For staining, sections were first rehydrated with DPBS and then stained with Fast Green solution (AppliChem, Germany) and 0.1% Safranin-O.

### 2.9 Language Editing

Grammarly and ChatGPT (OpenAI) were used to improve the grammar and make the manuscript more concise. No content was generated by AI; all scientific claims, results, and conclusions were developed by the authors.

## 3 Results

### 3.1 Bioink Preparation and Demonstration of Printability

Although the mixing of two components may appear straightforward, in the case of carboxylated agarose and collagen (type 1), their opposing gelation behavior necessitates precise handling, as they could interfere with each other and even phase separate. The presented method requires careful monitoring of the temperature at each step. CA was dissolved above 80 °C and then kept above its gelation temperature of approximately 35 °C, which varies slightly depending on the degree of carboxylation. The collagen solution was stored at 4 °C, since gelation might occur near room temperature. For optimal fibrillogenesis, the acidic pH of the collagen solution must also be neutralized; in our system, this was achieved automatically using DPBS as the solvent.

To evaluate the successful mixing and subsequent 3D printing performance, we chose geometries that feature small structures, overhanging regions, and a high height-to-weight ratio. These aspects can prove fidelity, a reliable gelation, and sufficient layer-to-layer adhesion, as well as the capability to sustain its weight under a shifting center of gravity. A three-legged arch with a single perimeter thickness was chosen as a representative test model, as previously described (Figure 2A,B).^23^ Despite containing nearly 50% collagen solution, the model was printed with high fidelity (Figure 2C). Individual layers were visible, as expected from extrusion-based printing, but the construct retained its form, and the three struts successfully converged in the center, demonstrating the capability of the bioink to preserve the favorable rheological properties of the CA. Importantly, the printed construct could be handled and transferred for imaging without structural failure.

**Figure 2:**
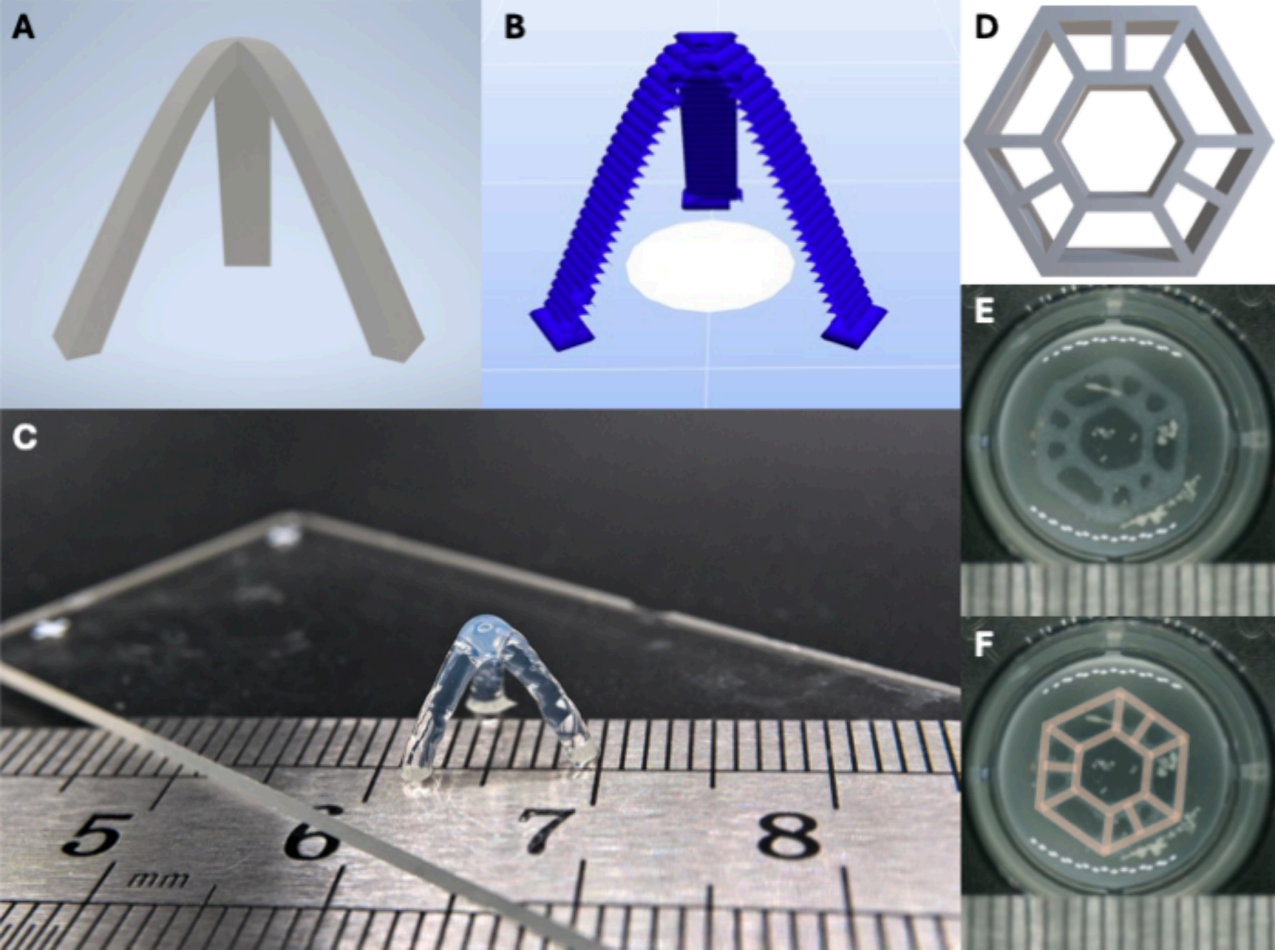
Printability demonstration of the hybrid bioink composed of CA with 4 mg/mL collagen type 1. (A–C) Three-legged arch. (A) Computer-aided design model, (B) corresponding gcode showing the layer-by-layer toolpath with single-wall thickness, and (C) photograph of the printed construct. (D–F) Hexagonal disk used for biological assays. (D) Computer-aided design model, (E) photograph of the printed cell-laden construct after 1 week of culture, and (F) overlay of the CAD design on the printed construct.

A hexagonal disk with cutouts of different sizes was selected as the printing model for biological experiments, since the priority was reproducibility and practicality. Additionally, the openings were incorporated to promote nutrient diffusion (Figure 2D). This model, while not as mechanically demanding as the three-legged arch, demonstrates submillimeter accuracy while being practical in a biological context. Since biological assays require multiple identical replicates, a semiautomated workflow was implemented: The gcode was modified to print 10 constructs sequentially at the same position, with a 10-s pause in between each construct. During each pause, the construct was removed with a spatula and transferred directly into a well plate. Using this approach, the collagen-CA bioink, laden with cells, could be transferred immediately after printing without structural damage (Figure 2E). Comparison of the printed constructs to the original design confirmed that the hybrid bioink could be printed with dimensional accuracy (Figure 2F).

### 3.2 Rheological Characterization

A key limitation in preparing collagen-based bioinks is the solubility of type 1 collagen in aqueous solvents. The commercially available solutions typically reach up to 10 mg/mL, though the exact value varies from batch to batch. In our case, the stock solution was 8.2 mg/mL, which restricted the maximum achievable concentration in the hybrid bioink to 4 mg/mL (nearly a 1:1 mixture with CA). Higher concentrations are generally difficult to obtain because collagen type 1 has a strong tendency to form fibrils under neutral pH, and even with concentrated acetic acid, its solubility is limited. The concentration of the CA was fixed at 9.5%wt to enable comparison with earlier studies, as higher concentrations cannot be sterilized via syringe filtration. When mixing CA with type 1 collagen to attain 4 mg/mL, a potential concern was that the reduction in CA content might compromise printability by impairing the rheological properties. Since the rapid gelation around 35 °C underpins its excellent printability, we conducted temperature sweeps and determined the storage modulus (*G*′) and loss modulus (*G*″) to assess whether gelation behavior was altered, pure collagen type 1 in DPBS (4 mg/mL) was used as control (Figure 3). The gelation point was determined as the crossover point of *G*′ and *G*″, where the temperature and modulus values were plotted (Figure 3, central radar plot).

**Figure 3:**
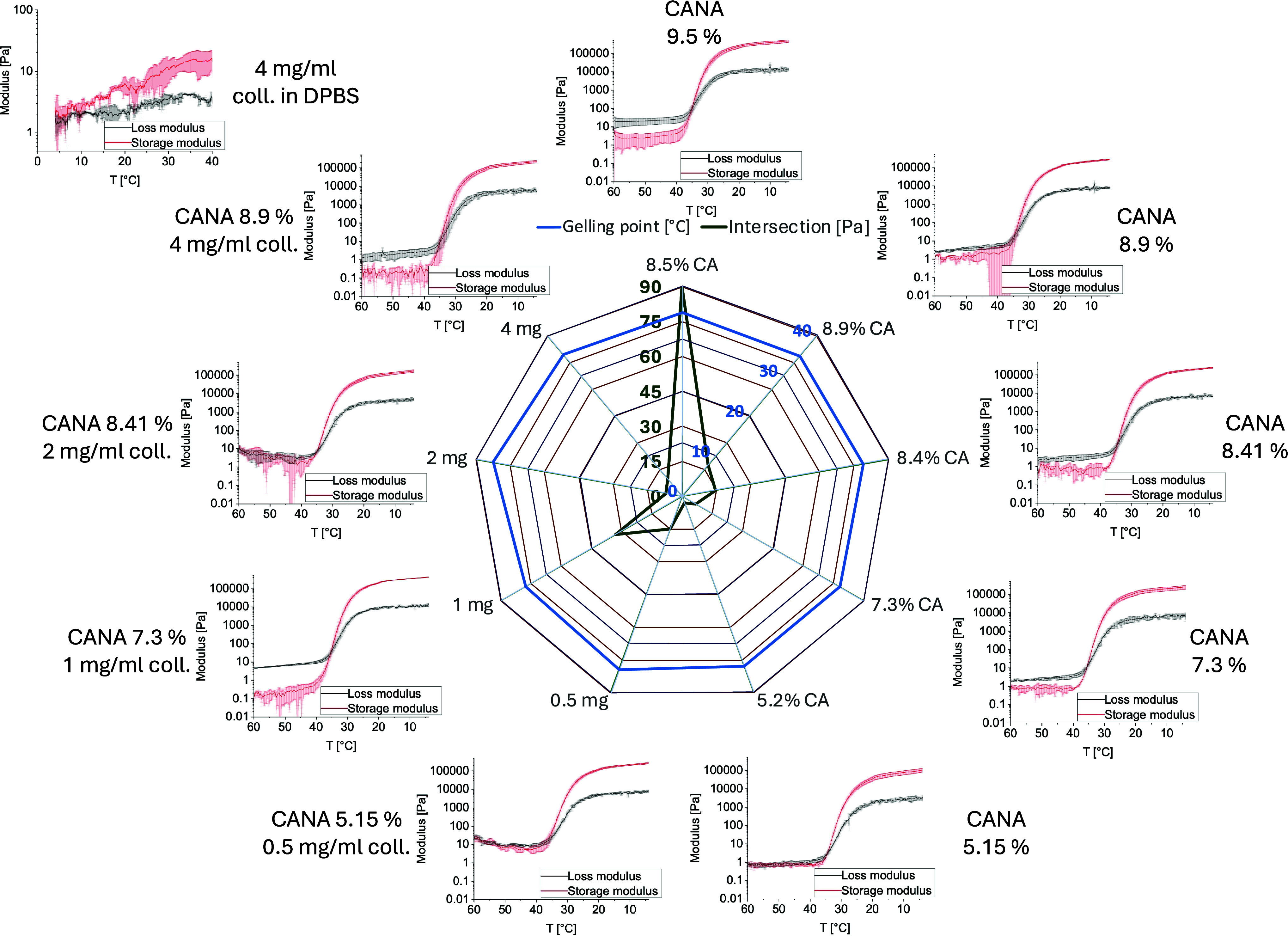
Rheological analysis of carboxylated agarose hydrogels with varying concentrations of collagen type 1: Temperature sweep curves for each formulation showing *G*′ (red) and *G*″ (gray). Data represent the average of three replicates with standard deviation. (Central radar plot) Comparison of key rheological parameters. The gelation temperature (blue) and corresponding *G*′/*G*″ crossover modulus at the gelation point (green). The rheological behavior of pure collagen at a concentration of mg/mL in DPBS (control) is shown in the adjacent plot on the upper left-hand corner.

The temperature sweep experiments demonstrated that the gelation mechanism of CA was preserved in all formulations, with gelation temperatures remaining consistent around 35.2 ± 0.6 °C. After gelation (measured at 6 °C), *G*′ and *G*″ values remained within the expected range (*G*′ ≈ 250.2 ± 107.7 kPa and *G*″ ≈ 7.4 ± 3.1 kPa). This suggests that the hybrid bioink retained most of its mechanical properties despite the reduction of CA by nearly 50%. Around the gelation point, however, a decrease in mechanical strength was observed. In thermogelling systems, this behavior is a known challenge as many materials lack sufficient strength immediately after extrusion.^24^ If constructs are not cooled rapidly enough, this reduction could result in spreading, poor resolution, or even the collapse of printed features. In our case, the use of a water-cooled print bed was sufficient to mitigate this issue, provided the print volume was less than 10 mL. Finally, the addition of collagen type 1 produced a minor increase in mechanical strength near the gelation point. Nevertheless, this effect is secondary to the influence of the carboxylation degree of agarose, which is known to tune stiffness across several orders of magnitude.^17^ The gelation of pure collagen type 1 occurred gradually over time with an increasing rate as temperature increased, rather than at a distinct temperature, supporting the theory of slow fibrillogenesis during printing with its final network formation happening postprinting, after the construct is transferred to the incubator. Additionally, *G*′ and *G*″ of a 4 mg/mL solution were orders of magnitude lower than mixtures with CA or CA alone, which proves clearly that CA provides the mechanical properties of the hybrid bioink.

While rheological analysis demonstrated that the gelation temperature and postgelation stiffness of the hybrid bioink were preserved, despite the dilution of the CA fraction, these measurements alone cannot reveal whether collagen type 1 and CA form a uniform network or undergo phase separation. To address this structural question, scanning electron microscopy was utilized to directly visualize the microstructure of the hybrid bioink.

### 3.3 Scanning Electron Microscopy

When combining two hydrogel-forming polymers, a key concern is whether they mix into a uniform network or undergo phase separation. We hypothesized that collagen could form a uniform network with CA in one of two ways: Either by forming a fibrillar network slowly enough to interpenetrate with the CA matrix after extrusion or by initially gelling at elevated temperatures during mixing and subsequently redissolving upon cooling to 6 °C after extrusion, thereby redistributing uniformly within the ink. This hypothesis was supported by visual inspection during printing, as no irregularities or aggregates were observed in the constructs, even under light microscopy. To visually examine the microstructure, scanning electron microscopy (SEM) was employed to determine whether the hybrid hydrogel underwent phase separation or mixed into a uniform network. Samples were prepared as pure CA, pure collagen (type 1), and a 1:1 CA-collagen mixture. Following critical point drying, the hydrogels were freeze-fractured, sputter-coated, and imaged immediately (Figure 4A).

**Figure 4:**
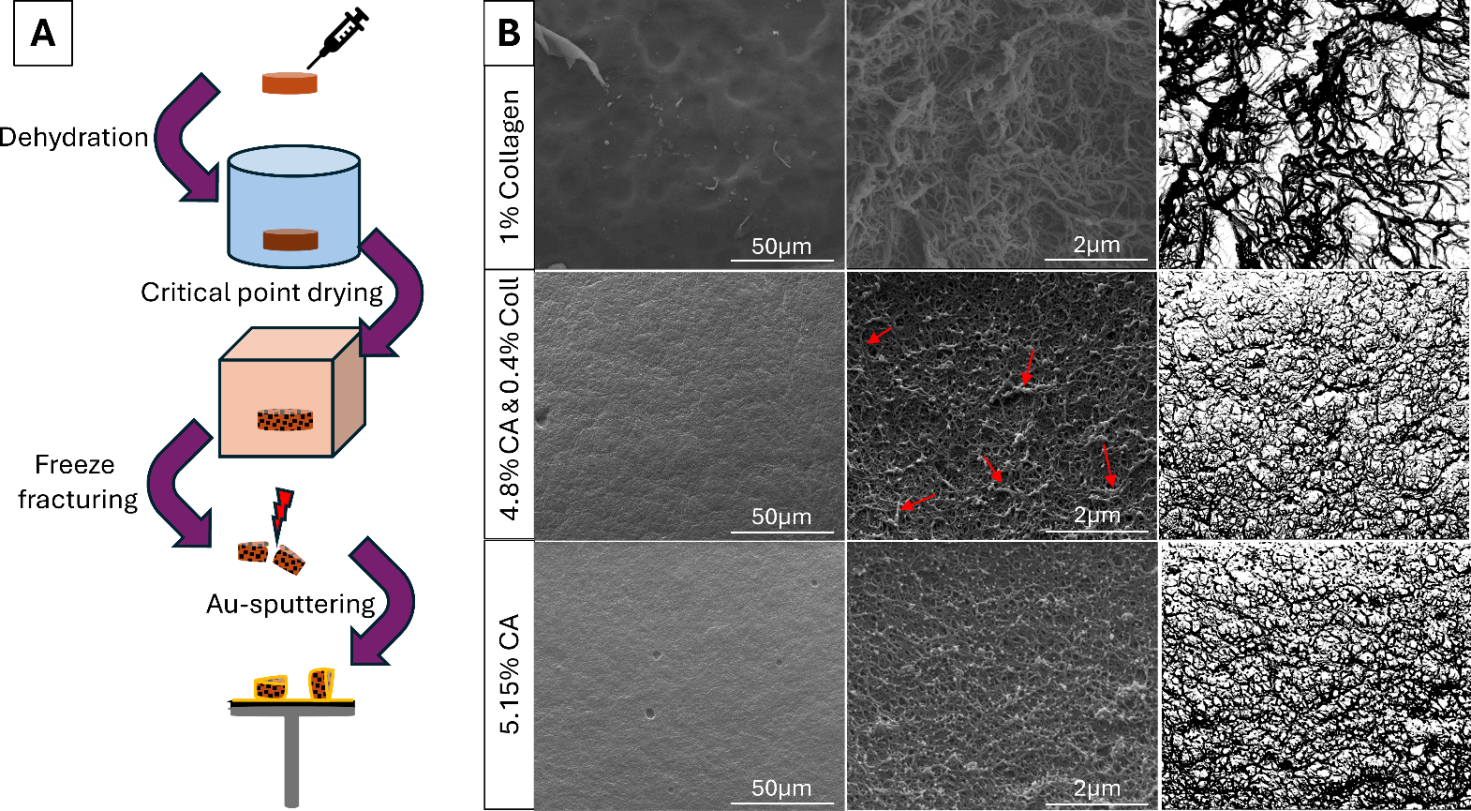
Scanning electron microscopy images of critical-point dried hydrogels. (A) Schematic workflow illustrating the preparation of hydrogel samples for SEM imaging. (B) Representative SEM images at low (left) and high (center) magnifications with threshold depiction of the high magnification (right) of pure type 1 collagen (top row), collagen with CA (middle row), and pure CA (bottom row). Red arrows indicate the presence of collagen fibrils within the CA network.

Upon neutralization and warming, solubilized type 1 collagen undergoes fibrillogenesis, resulting in the self-assembly of long, randomly oriented fibrils. In our samples, these fibrils could be visualized and exhibited an average diameter of 44 ± 22 nm, consistent with previously reported values (Figure 4B, upper row).^25^ By contrast, CA formed a porous, sponge-like network with relatively uniform pore structures (Figure 4B, bottom row). The hybrid hydrogel displayed a morphology in which the sponge-like CA network dominated but was regularly interrupted by fibrillar structures similar to collagen type 1 (indicated with red arrows in Figure 4B, middle row). The limiting factor, the collagen stock solution concentration, resulted in the structure being primarily composed of CA, with only a small mass portion of collagen. That explains why the overall microstructure was CA-dominated. Nevertheless, the presence of collagen fibrils integrated throughout the matrix, including at the surface, confirms that collagen was uniformly distributed within the hybrid gel. Importantly, no regions resembling the morphology of pure collagen gels were detected, and no evidence of phase separation was observed. The integrated collagen fibrils suggest that the bioink is likely to support cell adhesion, as the collagen is accessible within the entire network and not confined to isolated domains.

### 3.4 Attachment of Cells on Cast Gels

Since the primary purpose of adding type 1 collagen to the already established CA bioink was to improve biological interaction, an attachment assay was performed. Human hepatocytes (HepG2) were seeded onto cast hydrogels in 48-well plates and imaged periodically to assess morphology and attachment behavior (Figure 5A). At a minimal concentration of 1 mg/mL, hepatocytes displayed improved adhesion, forming interconnected structures instead of the spherical aggregates observed on pure CA. When exchanging medium or washing with PBS, cells on pure CA and on lower collagen concentrations were largely washed off, whereas those on 1 mg/mL collagen remained attached. At 0.5 mg/mL collagen, the size of spherical aggregates was reduced, and their number increased, suggesting that individual cells may have partially adhered and thereby acted as nucleation points for small clusters. Increasing collagen concentration beyond 1 mg/mL (data not shown) did not substantially improve attachment, suggesting that ∼1 mg/mL represents a minimal threshold for HepG2 adhesion in this system, but also that the maximum concentration of 4 mg/mL is not sufficient to form confluent monolayers. This is likely due to the limited number of collagen fibrils available for cell attachment, as suggested by SEM observations. In sum, we could show that the small amount of collagen in the hybrid gels provides sufficient cues for adhesion, but not for confluent cell coverage.

**Figure 5:**
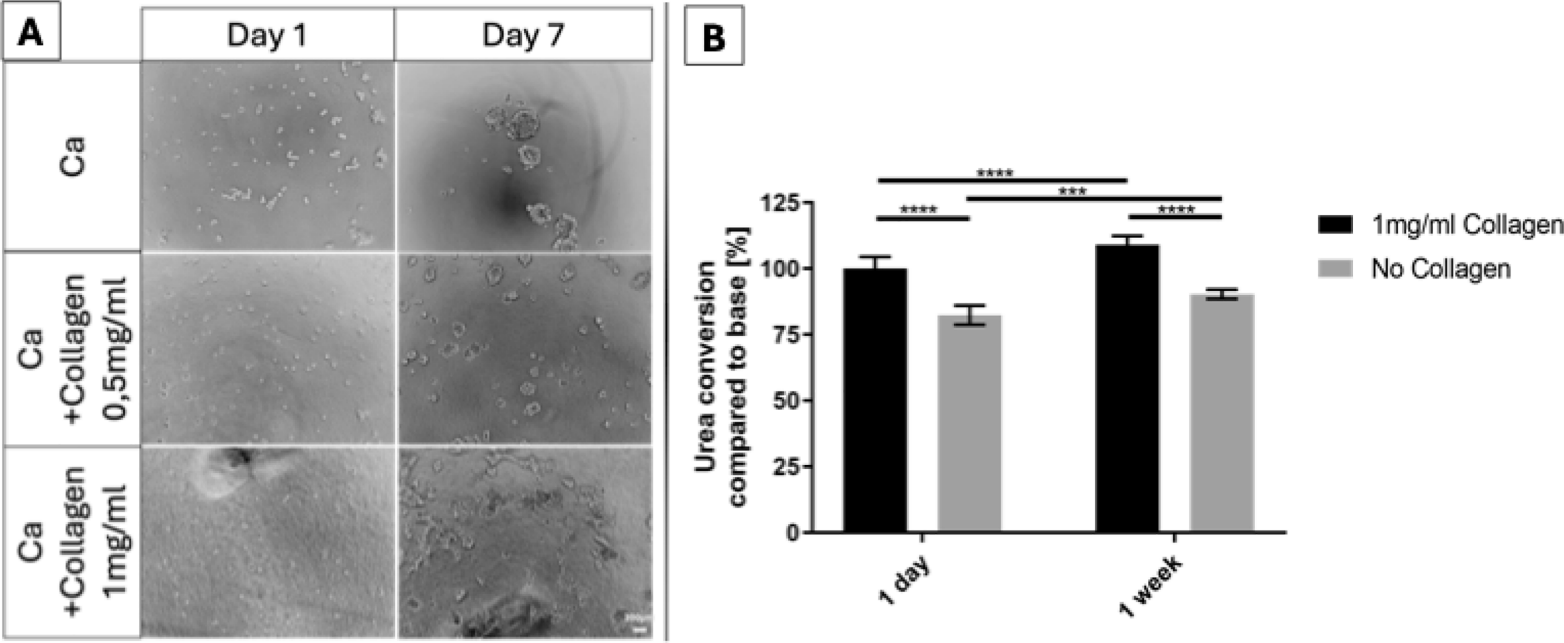
Cell attachment and urea conversion assay of HepG2 cells on and in CA hydrogels with and without collagen type 1. (A) Representative phase-contrast microscopy images of HepG2 cells cultured on top of cast hydrogels with varying concentrations of collagen type 1; scale bar = 200 μm (applies to all images). (B) Quantification of hepatic metabolism via blood urea nitrogen assay. HepG2 cells were bioprinted in CA with or without 1 mg/mL collagen type 1. Data were normalized to the collagen-containing condition at day 1 and adjusted to construct weight. Statistical analysis was performed using one-way ANOVA (GraphPad Prism 9); *n* = 9, error bars represent standard deviation.

To assess the functional impact of collagen type 1 incorporation on hepatocytes, a blood urea nitrogen (BUN) assay was performed, which measures the conversion of ammonia to urea. Based on the attachment study, 1 mg/mL collagen was selected for comparison with pure CA (Figure 5B). As expected, hepatocytes in collagen-containing gels exhibited significantly higher urea conversion rates after both 1 day and 1 week compared to pure CA. The fact that conversion increased significantly over time in both conditions suggests that neither CA nor our hybrid bioink affects cells adversely and allows growth.

### 3.5 Collagen-Enriched Bioink Increases Proliferation and ECM Deposition in Human Nasal Chondrocytes

To evaluate the influence of collagen type 1 incorporation on cell survival and extracellular matrix (ECM) formation in 3D, primary human nasal chondrocytes were bioprinted with either 1 or 4 mg/mL collagen, alongside collagen-free controls. Cell number was assessed via DNA content, while ECM deposition was quantified through glycosaminoglycan (GAG) assays and stainings (Figure 6A). DNA content decreased over 1 week in the collagen-free gels, indicating a decreasing number of cells (Figure 6B). This is likely due to the cells not being able to attach to their surrounding matrix. Cells are known to stop proliferating and eventually die when they remain unattached. In contrast, both collagen-containing conditions showed increased DNA content, consistent with increasing cell number, suggesting proliferation. Comparable to the results of the attachment assay, collagen type 1 incorporation provided adhesion sites and thereby facilitated cell survival and even expansion within the hybrid hydrogel. GAG production increased in all groups, demonstrating the inherent potential of the CA-based hydrogel for chondrogenic applications (Figure 6C). The highest GAG increase was observed in the 1 mg/mL condition, whereas the 4 mg/mL condition showed a lower amount of GAG than even the collagen-free control. This suggests that a low collagen content may be best for balancing proliferation and matrix production, while a higher concentration seems to induce a more proliferative phenotype with reduced matrix deposition. Although these findings indicate a concentration-dependent effect, further studies with a broader range of collagen contents need to be undertaken to verify and determine the optimal concentration for specific behavior. Histological staining with safranin O supported the quantitative data (Figure 6D): GAG deposition was visualized in the pericellular regions of all groups, visible as darker ring-shaped areas around cells. Although interpretation was complicated by background staining of CA, the 1 mg/mL collagen group exhibited the most intense GAG deposition, while the 4 mg/mL group showed reduced staining. Verification of the proliferation in the collagen-containing gels, as concluded from the DNA assay, was not attempted, since cell numbers vary strongly within areas of a single gel.

**Figure 6:**
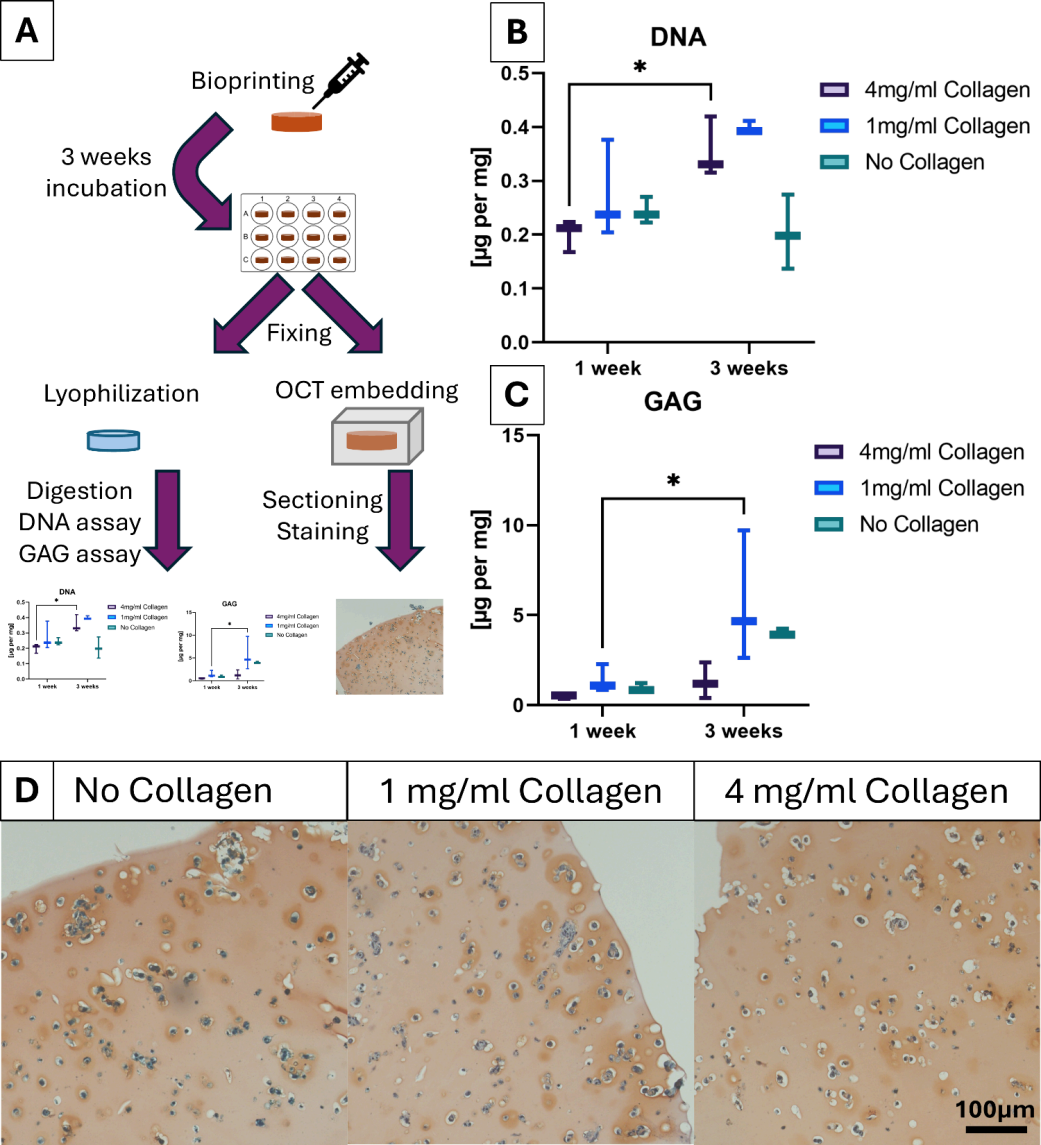
Evaluation of proliferation and ECM deposition of human nasal chondrocytes in 3DBP hybrid hydrogels. (A) Schematic overview of the workflow from 3D bioprinting to analysis via DNA/GAG assay and histology. (B) Quantification of DNA content in bioprinted constructs after 1 and 3 weeks. Constructs were printed with CA containing varying concentrations of collagen type 1 and normalized to print weight. (C) GAG quantification from the same constructs, normalized to print weight. (D) Safranin O staining of representative histological sections after 3 weeks of culture; scale bar = 100 μm (applies to all images). Statistical analysis for panels (B, C) was performed using one-way ANOVA (GraphPad Prism 9); *n* = 3, error bars represent standard deviation.

Taken together, these experiments demonstrate that even low levels of collagen type 1 enable proliferation and ECM deposition in the otherwise biologically inert CA hydrogel.

## 4 Discussion

This study demonstrates that collagen type 1, an ECM protein with important functions in cell fate and function, can be incorporated into carboxylated agarose (CA) bioinks straightforwardly, creating a hybrid hydrogel that benefits from both components without compromising on either. The strategy presented here is not limited to CA-collagen mixtures but can be applied to other hydrogel-forming polymers as well, thus providing a promising example for hybrid bioink design in the future.

A key finding is that CA and collagen type 1, despite their contrasting gelation conditions, can be combined into a uniform and printable hybrid hydrogel. The material retained the favorable rheological properties of CA—rapid gelation, sufficient stiffness for structural fidelity, layer-to-layer adhesion, and low printing pressure—while gaining biological functionality from type 1 collagen. Scanning electron microscopy confirmed that no phase separation occurred. Instead, collagen fibrils were evenly distributed within the sponge-like CA network. This integration helps to explain enhanced cellular attachment observed in biological assays in a microstructural way. To further enhance this visualization, we then combined CA with FITC-conjugated collagen type 1 and imaged the cast hybrid gels under a fluorescent microscope. The homogeneously distributed fluorescent signal clearly confirmed that the collagen was evenly integrated throughout the CA network in both coverslipped gel drops (Figure 7A,B) and cryosectioned bulk samples (Figure 7C). One limitation was identified in the low concentration of commercially available collagen type 1 solutions, typically between 8 and 12 mg/mL, which limited the achievable final concentrations to 4 mg/mL. Alternatives such as atelocollagen or enzymatically shortened collagen fibers, with greater solubility, could be ways to overcome this bottleneck, as well as a recently published stratgey where *N*-hydroxy succinimide-modified poly­(acrylic acid) (PAA), was used to synthesize collagen-PAA conjugates that showed solubility in water at a neutral pH.^26^ An additional consideration for the translation of this hybrid bioink is the stability of the double-network in vitro and in vivo. While the in vivo response is difficult to predict, it is known that CA is largely resistant to hydrolysis and enzymatic degradation, whereas collagen is degraded over time by exogenous collagenase or matrix metalloproteinases. In vitro, the degradation of collagen would depend in cell culture media (serum or no serum) and the cell type, as many cells secrete proteases. In general, the degradation of collagen would provide more space for the deposition of matrix by cells. In vivo, CA has been shown to be resorbed through a mechanism that is yet to be fully elucidated, but most likely through macrophage activity, undergo vascularization and support matrix deposition by mesenchymal cells.^18^ While, all of this points to the potential of CA-Collagen hybrid bioink undergoing remodeling and degradation in vivo, nonetheless, future studies will be required to investigate whether that degradation mismatch leads to the cells creating their own microenvironment/ECM, substituting the collagen, over time, and the stability of printed constructs.

**Figure 7:**
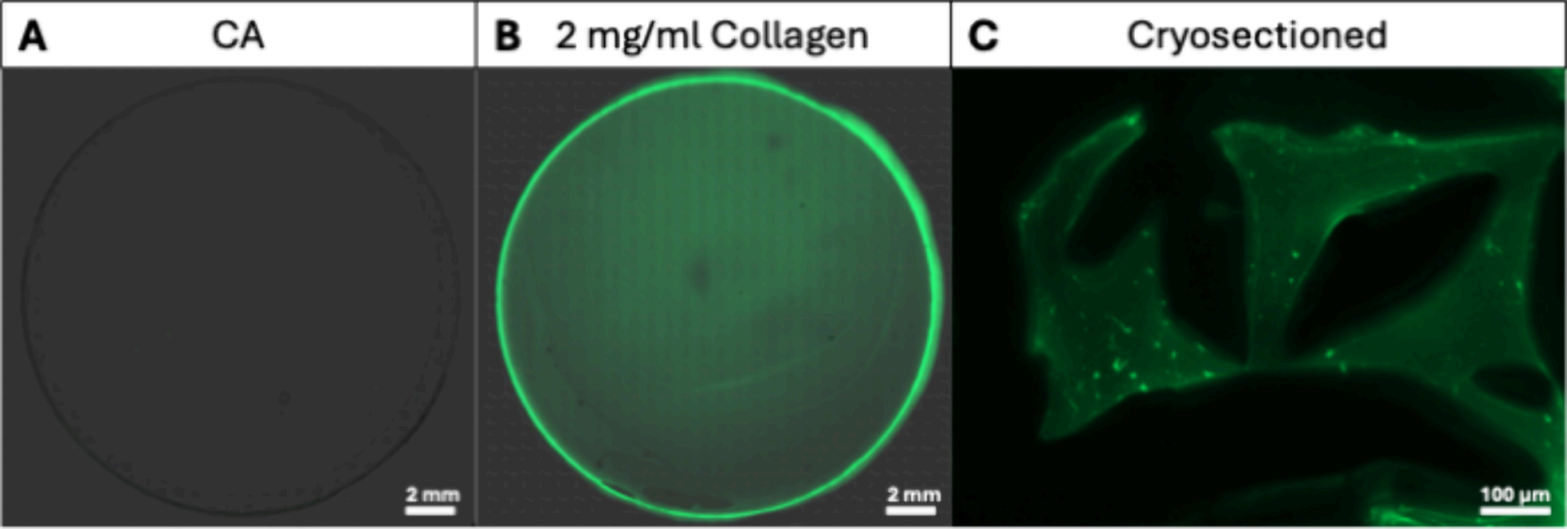
Fluorescent microscopy of CA mixed with FITC-conjugated collagen type 1. (A) Fluorescent microscopy/brightfield-overlay image of 1 drop of CA with no collagen, gelled on a microscopy slide with a coverslip. (B) Fluorescent microscopy/brightfield overlay image of 1 drop of CA with 2 mg/mL FITC-collagen type 1, imaged under identical conditions. (C) Representative fluorescent microscopy image of cryosectioned (10 μm) bulk samples of CA with 2 mg/mL FITC-collagen type 1. Bright green regions are fibrils of collagen, presumably formed through fibrillogenesis.

The biological results demonstrate that 1 mg/mL collagen is sufficient to transform an otherwise bioinert CA hydrogel into a bioink supportive of cell adhesion, proliferation, and increased metabolic activity. Hepatocytes attached and spread on casted gels containing collagen, whereas they formed spheroid-like agglomerates and detached on collagen-free CA gels. When bioprinted in the hybrid ink, hepatocytes exhibited significantly increased urea conversion—a functional marker of liver metabolism^27^—while human nasal chondrocytes showed increased proliferation and ECM deposition. Interestingly, 1 mg/mL collagen resulted in the strongest increase in GAG production, while 4 mg/mL led to low GAG deposition, but the highest proliferation. This observation indicates that collagen type 1, and hence cellular attachment, also influences hNC phenotype, either toward proliferative or metabolic states.

## 5 Conclusions

Together, these findings extend the portfolio of CA-based bioinks. Previous approaches have incorporated gelatin in soluble or microparticle form to increase bioactivity. Here, collagen type 1 and CA were integrated with a complementary strategy to enable cell-matrix interactions. The modularity of all three approaches suggests that combinations of these formulations may be utilized to meet the biological requirements of challenging applications while maintaining the favorable rheological and mechanical properties of CA. Beyond biomedical tissue engineering, the edible and naturally derived nature of both components opens possibilities in food biotechnology, such as engineered meat. Because CA remains edible after carboxylation and collagen is already a major component of natural meat, CA-collagen bioinks could serve as structured, printable matrices for alternative meat production.

## ABBREVIATIONS

3DBP: 3D bioprinting
ECM: extra cellular matrix
CA: carboxylated agarose
NA: native agarose
SEM: scanning electron microscopy
CPD: critical point drying
CAD: computer aided design
DPBS: Dulbecco’s phosphate-buffered saline
DMEM: Dulbecco’s Modified Eagle medium
GAG: glycosaminoglycan
DNA: deoxyribonucleic acid
NC: nasal chondrocyte
ANOVA: analysis of variance
PAA: poly­(acrylic acid)
